# Nuclear epidermal growth factor receptor as a therapeutic target

**DOI:** 10.37349/etat.2023.00156

**Published:** 2023-08-30

**Authors:** Benjamin Atwell, Pavani Chalasani, Joyce Schroeder

**Affiliations:** Institute of Experimental Endocrinology and Oncology “G. Salvatore”-National Research Council (IEOS-CNR), Italy; ^1^Department of Molecular and Cellular Biology, University of Arizona, Tucson, AZ 85721, USA; ^2^Department of Medicine, University of Arizona, Tucson, AZ 85721, USA; ^3^University of Arizona Cancer Center, Tucson, AZ 85721, USA; ^4^Bio5 Institute, University of Arizona, Tucson, AZ 85721, USA

**Keywords:** Triple negative breast cancer, epidermal growth factor, mucin-1, retrotranslocation

## Abstract

Epidermal growth factor receptor (EGFR) is one of the most well-studied oncogenes with roles in proliferation, growth, metastasis, and therapeutic resistance. This intense study has led to the development of a range of targeted therapeutics including small-molecule tyrosine kinase inhibitors (TKIs), monoclonal antibodies, and nanobodies. These drugs are excellent at blocking the activation and kinase function of wild-type EGFR (wtEGFR) and several common EGFR mutants. These drugs have significantly improved outcomes for patients with cancers including head and neck, glioblastoma, colorectal, and non-small cell lung cancer (NSCLC). However, therapeutic resistance is often seen, resulting from acquired mutations or activation of compensatory signaling pathways. Additionally, these therapies are ineffective in tumors where EGFR is found predominantly in the nucleus, as can be found in triple negative breast cancer (TNBC). In TNBC, EGFR is subjected to alternative trafficking which drives the nuclear localization of the receptor. In the nucleus, EGFR interacts with several proteins to activate transcription, DNA repair, migration, and chemoresistance. Nuclear EGFR (nEGFR) correlates with metastatic disease and worse patient prognosis yet targeting its nuclear localization has proved difficult. This review provides an overview of current EGFR-targeted therapies and novel peptide-based therapies that block nEGFR, as well as their clinical applications and potential for use in oncology.

## Introduction

Discovered in 1965 by Cohen [1], the epidermal growth factor receptor (EGFR) is a member of the transmembrane tyrosine kinase human EGFR (HER)/ErbB family. The HER family of receptors are potent drivers of growth, proliferation, and migration [2] and consist of EGFR, ErbB2, ErbB3, and ErbB4. While essential for the development and maintenance of normal epithelial tissue, changes to EGFR biology including overexpression/amplification [3, 4], mutation [5, 6], and altered trafficking [7, 8], allow aberrant signaling and functions leading to cancer and other diseases. As a transmembrane tyrosine kinase, therapeutic interventions for EGFR-driven cancers have largely focused on targeting via monoclonal antibodies and tyrosine kinase inhibitors (TKIs). These therapies have shown some success, as in EGFR overexpressing head and neck [9], and lung adenocarcinoma [10]. Although these treatments are initially effective, the majority of tumors eventually acquire resistance via several mechanisms [11].

### Monoclonal antibodies

**Figure 1:**
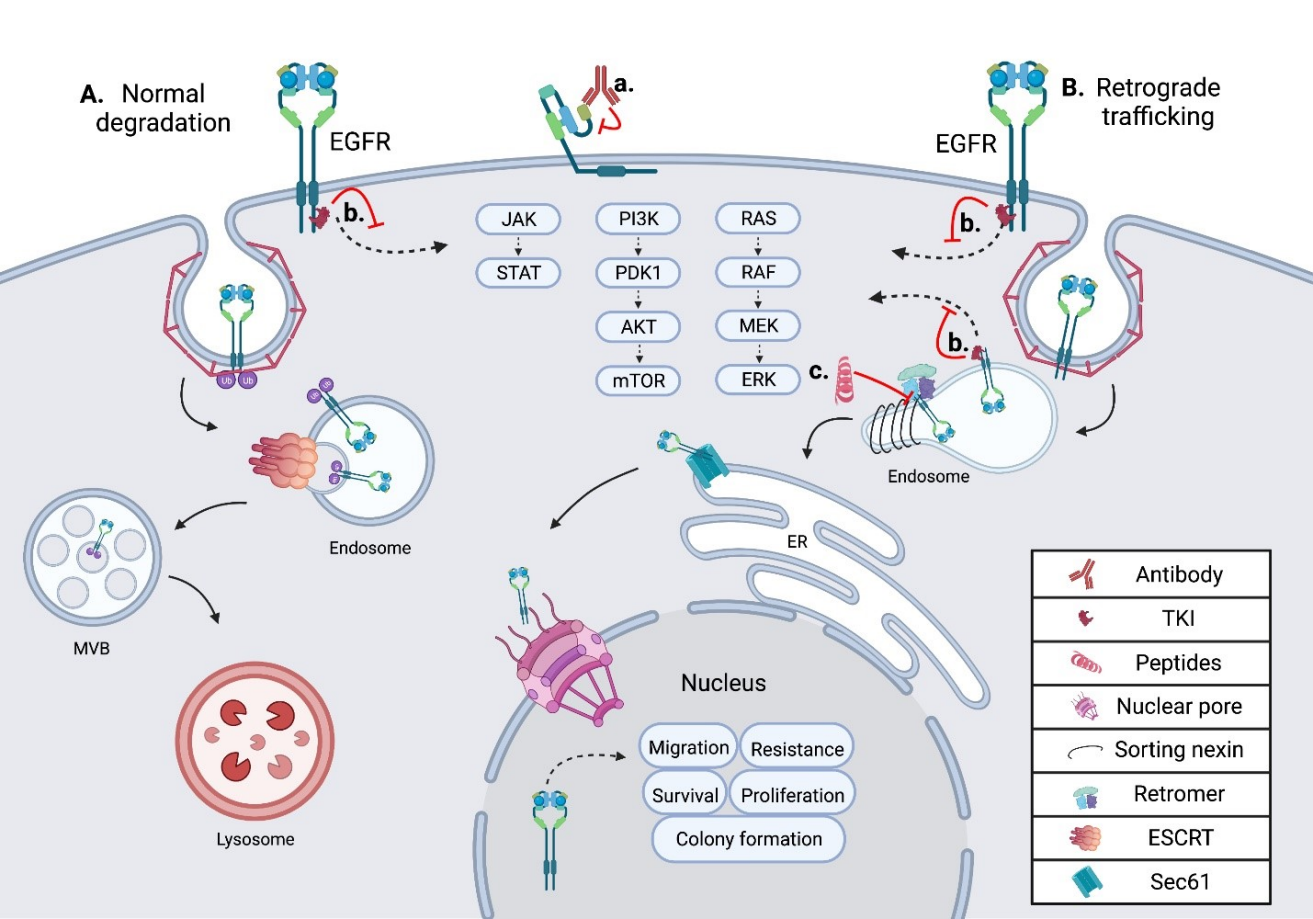
Schematic showing the intracellular trafficking and pharmacological inhibitors of EGFR. (A) Shows the internalization and degradation of EGFR, seen in normal polarized epithelium; EGFR is first internalized through clathrin-mediated endocytosis, then undergoes trafficking through the endosomal network before being degraded in the lysosome; (B) the retrograde trafficking of EGFR is depicted, beginning with clathrin-mediated endocytosis; EGFR is then bound by sorting nexins (SNXs) and the retromer within the endosome, inducing further retrograde trafficking to the nucleus; within the nucleus EGFR drives oncogenic pathways through several mechanisms; (a) an EGFR targeted monoclonal antibody is depicted binding EGFR at the cell surface, inhibiting ligand binding and subsequent activation; (b) an EGFR specific TKI is depicted inhibiting EGFR signal transduction from the plasma membrane and the endosome in both normal cells and tumor cells; (c) the EGFR retrograde trafficking inhibitor cSNX1.3 is shown blocking the interaction of EGFR and cSNX1.3 which blocks the nuclear accumulation of EGFR. AKT: protein kinase B; cSNX1.3: capped sorting nexin peptide 1.3; ER: endoplasmic reticulum; ERK: extracellular signal-regulated kinase; ESCRT: endosomal sorting complex required for transport; JAK: Janus kinase; MEK: mitogen-activated protein kinase kinase; mTOR: mechanistic target of rapamycin; MVB: multivesicular body; PDK1: 3-phosphoinositide-dependent kinase 1; PI3K: phosphatidylinositol 3-kinase; RAF: rapidly accelerated fibrosarcoma; RAS: rat sarcoma virus; Sec61: transmembrane translocon channel; STAT: signal transducer of activators of transcription

Cetuximab was the first EGFR targeted therapeutic antibody to gain FDA approval in 2004 and acts by competitively inhibiting the ligand binding pocket to prevent activation (Figure 1) [12]. In phase 3 studies, when given in combination with chemotherapy or radiation, the inclusion of cetuximab has increased median overall survival by ~3 months for colorectal cancer [13], ~5 months in head and neck [14], and ~8 months for non-small cell lung cancer (NSCLC) [15]. Since then, several other antibodies have been developed including panitumumab, nimotuzumab, necitumumab, and zalutumumab [16, 17]. These antibodies have shown efficacy in clinical trials for colorectal, head and neck, non-small cell lung, and biliary cancers. Most of these antibodies act by blocking the EGFR ligand binding pocket. However, nimotuzumab induces natural killer (NK) cell activation and T-cell expansion in addition to blocking ligand binding. This immune activation allows nimotuzumab to be more effective than cetuximab despite having a lower binding efficiency to EGFR [18]. Despite promising efficacy in multiple tumors, these antibodies have had little effect in triple negative breast cancer (TNBC) [19], even though these cancers express high levels of EGFR.

### Conjugated antibodies

Antibody-drug conjugates (ADCs) targeting EGFR have also shown promise in treating certain cancers. One of the first ADCs was an EGFR targeted antibody (mAb108), which was conjugated to the DNA intercalating agent, doxorubicin, in 1989 [20]. This ADC, mAb108-dextran-doxirubicin, showed a significantly improved anti-tumor effect when compared to free doxorubicin in mouse xenografts. Since their introduction, several next generation ADCs have been developed which use more tumor specific antibodies and stronger payloads [21]. Depatuxizumab mafodotin (ABT-414) is an EGFR targeted ADC currently in phase 3 clinical trials for glioblastoma [22]. ABT-414 uses the EGFR targeted mAb806 which has a higher affinity for EGFRvIII and greater efficacy against glioblastoma than cetuximab [23]. This antibody is conjugated to the anti-microtubule agent monomethyl auristatin F (MMAF) which is highly toxic as a single agent yet becomes tolerable with the tumor specificity granted by the conjugated antibody. In the phase 2 clinical trial for ABT-414 in glioblastoma patients, a hazard ratio of 0.66 was found for ABT-414 plus temozolamide compared to the temozolamide alone control arm [24]. Another ADC, M1231, uses a bispecific antibody that binds EGFR and mucin-1 (MUC1), proteins that are isolated to separate membrane domains in healthy tissue and colocalize during tumorigenesis [25]. A recent phase 1/1b clinical trial of the anti-EGFR antibody-monomethyl auristatin E, MRG003, found manageable toxicity with some efficacy in head and neck and nasopharyngeal carcinoma [26].

Another group has generated an antibody conjugate for combination therapy by creating a multilayered sphere called, anti-EGFR-PTX-TCS-GNS. The layers from the innermost are a silica sphere, gold shell, thiol chitosan, paclitaxel, and EGFR directed antibodies [27]. This complex drug simultaneously optimizes several therapies to create a first-in-class combinatorial therapeutic. Tumor directed gold particles increase the efficacy of radiotherapy by absorbing the near-infrared (NIR) wavelengths within the tumor, increasing the necrotic effect [28]. Thiol chitosan is a biopolymer that is nontoxic and biodegradable, as it absorbs heat from the NIR waves it melts releasing the paclitaxel payload.

### Nanobodies

Nanobodies are camel-derived antibodies that lack the antibody light chain. One of the first successful nanobodies targeted EGFR to block epidermal growth factor (EGF) binding and EGFR phosphorylation and showed promised in xenograft models, primarily the A431 xenograft model [29]. Since their initial development, nanobodies have become increasingly complicated and effective [30]. Shortly after the first EGFR targeted nanobody was created, the same group created several bivalent (2 conjugated nanobodies which bind the same epitope) and biparatopic (2 conjugated nanobodies which bind different epitopes of the same protein) nanobodies and tested their efficacy again in A431 xenografts [31]. They found that while bivalent nanobodies had a modest increase, biparatopic nanobodies showed a significant improvement in efficacy.

Subsequently, the creation of bispecific nanobodies (2 fused nanobodies which bind different proteins) with an αEGFR-αEGFR-αAlbumin nanobody, which binds albumin in the blood to reduce renal clearance and increase tumor delivery [32]. This nanobody showed greater tumor uptake and deeper tumor penetration than cetuximab. In 2021, a bispecific nanobody was created, using αEGFR nanobody (7D12) fused to αCD16 nanobody (C21) to bind NK cells [33]. The authors reported that in *in vitro* and *ex vivo* studies, this bispecific nanobody induced degranulation of NK cells.

### TKIs

TKIs are widely used in cancers with EGFR tyrosine kinase activating mutations as drivers, most effectively in lung adenocarcinoma, specifically NSCLC [34]. In NSCLC, EGFR TKIs significantly improve progression-free survival, as recently reviewed in [35]. EGFR-directed TKIs are designated into 3 generations of approved therapies with fourth generations currently being developed (Figure 1). First generation EGFR TKIs, including gefitinib and erlotinib, reversibly bind the ATP binding pocket within the kinase domain [36]. Second generation TKIs covalently bind the ATP pocket and showed improvement in PFS in head to head trials [37, 38]. However, these second-generation inhibitors also had slightly higher toxicity and side effects.

While first and second generation TKIs have shown significant clinical impact, the majority of tumors tend to acquire resistance to these drugs within a year [39]. While some tumors develop resistance through upregulation of other receptor tyrosine kinases (RTKs) or activating mutation in signaling intermediates, the majority of resistance arises with the EGFR T790M mutation [40]. This is a mutation to the ATP binding pocket that increase EGFRs affinity for ATP which outcompetes first and second generation TKIs [41]. A third generation EGFR TKI which overcome this mutation are currently available in clinic [42]. Osimertinib, an oral third generation irreversible EGFR TKI was shown in a pivotal phase 3 clinical trial to show superior efficacy to first and second generation EGFR TKIs when given as first-line therapy [43]. Similar to antibodies, these TKIs while having shown significant clinical impact in many cancers including; head and neck, NSCLC, and colon are ineffective in TNBC [44].

## Alternative trafficking as a mechanism of EGFR-driven oncogenesis

In TNBC, EGFR is frequently found amplified and overexpressed, but not mutated. In these tumors, it is observed in intracellular locations in addition to the plasma membrane [45]. Several protein interactions and regulatory events are required for altered intracellular localization of EGFR to occur, including the complexing of EGFR and MUC1 (model of trafficking; Figure 1). MUC1 is comprised of a C-terminal cytoplasmic tail, including a transmembrane and small extracellular domain, that is bound through cysteine bonds to the heavily *O*-glycosylated N-terminal extracellular domain [46]. In healthy epithelium, MUC1 is localized to the apical domain and acts as a protective barrier against infection and inflammation [47]. However, during cancer progression MUC1 regulation is altered, resulting in its overexpression and gain of several oncogenic functions [48]. Concurrent with MUC1 overexpression during tumorigenesis, a loss of cellular polarity across the epithelium occurs. Loss of polarity allows traditionally apical and basolateral localized proteins to interact, as is the case with EGFR and MUC1. The interaction of MUC1 and EGFR at the plasma membrane inhibits the ubiquitylation of EGFR [49]. This interaction increases the rate of receptor internalization into the early endosome (EE) and is paired with a loss in degradation. Under these conditions, EGFR is found to interact with the SNX, specifically SNX1 which directly binds EGFR and regulates its intracellular trafficking [50] (Figure 1).

The sorting nexin family is a diverse group of peripheral membrane proteins that are classified based on the SNX-phox homology (PX) domain [51]. The PX domain of the SNX family is a phosphatidylinositol 3-phosphate (PI3P) binding motif, with minimal affinity for PI2P, that allows recruitment to membranes throughout the endosomal network and plasma membrane [52]. Initial studies suggested that SNX1 enhanced the degradation of EGFR when expressed in cells that did not normally express SNX1 [50]. This is likely due to the ability of SNX to elongate tubule vesicles out of domains such as the EE [53]. SNX, including SNX1, can associate their cargo with the retromer complex, a heterotrimer of proteins required for sorting (Vps26, Vps29, and Vps35). This complex is involved in cargo recognition at the endosomal network and sorting for retrograde trafficking. Unlike the SNX which directly binds the membrane via its PX domains, the retromer has no membrane binding domains. Instead of directly binding the membrane, the retromer complex binds sorting proteins like Rab7 or the SNX, as they are recruited to late endosomes [54] (Figure 1). Together, SNX proteins recognize and bind cargo proteins such as EGFR, induce the formation of vesicles, associate with the retromer complex, and route cargo for retrograde trafficking.

**Figure 2:**
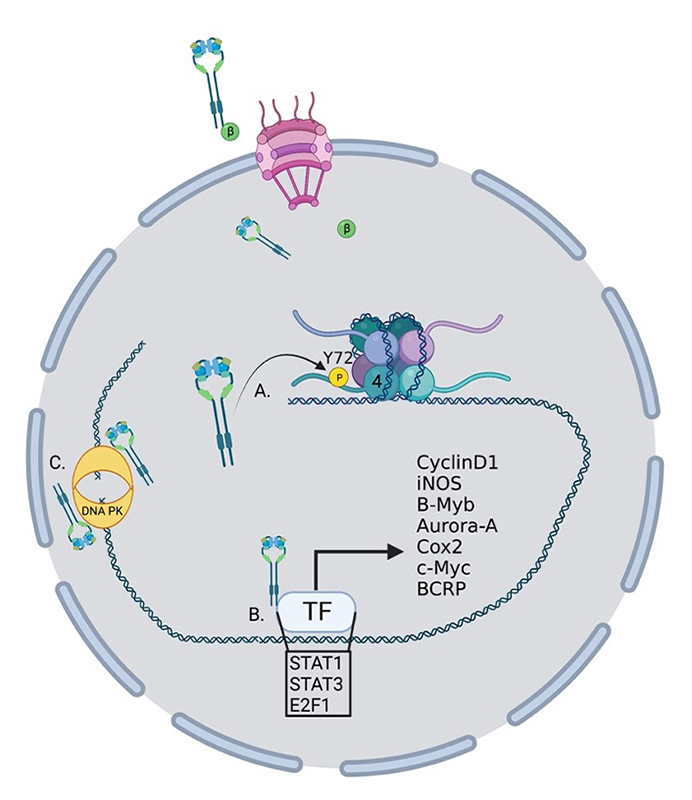
Oncogenic roles for nucelar EGFR. EGFR performs several functions within the nucleus to drive the transcription of oncogenes. (A) EGFR phosphorylates histone 4 Y72 which induces the recruitment of histone methyl transferaces and the methylation of histone 4 K20, enhancing DNA replication and repair; (B) EGFR interacts with several transcription factors to induce the transcription of a growing list of oncogenes; (C) EGFR interacts with DNA-dependent protein kinase (DNA-PK) to induce its translocation to the nucleus and activation of DNA repair. TF: transcription factor; P: phosphorylation; β: importin β

EGFR has several roles in the nucleus that can be either kinase dependent or more often kinase independent (Figure 2). While EGFR has no DNA binding domain, it can act as a co-transcriptional regulator for a growing list of oncogenes through associations with several nuclear proteins [55] (Figure 2). Nuclear EGFR (nEGFR) has been shown to interact with the transcription factors E2F1, STAT3, and STAT5 as well as RNA helicase A [56–59]. nEGFR can induce the expression of cyclinD1 [60], nitric oxide synthase (iNOS) [57], B-Myb [56], Aurora kinase A (Aurora-A) [58], cyclooxygenase-2 (COX-2) [61], c-Myc [62], and the breast cancer resistance protein, breast cancer resistance protein (BCRP) [63].

nEGFR has also been linked to resistance to radiotherapy through the activation of DNA repair pathways [64]. Infrared (IR) radiation can induce nuclear localization and interaction with DNA-PK and induction of DNA repair [65] and DNA repair can be eliminated by blocking retrograde trafficking and the nuclear accumulation of EGFR [66] (Figure 2). In another study, EGFR was shown to be recruited to the nucleus and aid in DNA repair in response to cisplatin treatment [67]. nEGFR has also been shown to modulate the epigenetic landscape by phosphorylating histone 4Y72 [68] (Figure 2). This phosphorylation increases the recruitment of the histone methyltransferases SET8 and SUV 4-20H leading to an increase in DNA transcription and repair.

The non-canonical trafficking and activities of EGFR may drive resistance to conventional therapies in several ways. The presence of EGFR within intracellular endosomes or the nucleus would prevent cell impermeable antibodies from reaching the receptor. Further, antibodies like cetuximab which act by competitively inhibiting the ligand binding pocket would not be effective as the ligand is already bound, and receptor activated and internalized [12]. Antibodies, like nimotuzumab, that act by recruiting immune cells would also be ineffective as they need to be on the cell surface to recruit immune cells [18]. TKIs on the other hand are freely cell permeable and inhibit EGFR activation through competitive inhibition of the ligand binding pocket. However, the noncanonical roles of nEGFR also evade TKI therapies as many of these roles may not involve EGFR’s kinase function. Instead, nEGFR can directly bind transcription factors within the nucleus and alter their DNA binding profiles to induce a novel suite of oncogenic protein expression. The shortcomings of conventional therapies to target intracellular and nEGFR highlight the need for new and different therapies that can inhibit the nuclear roles of EGFR in TNBC.

## Targeting EGFR retrograde trafficking

The nuclear accumulation of EGFR represents an ideal target for therapeutic intervention as it appears to rarely occur outside the context of cancer, yet drives tumor progression, therapeutic resistance, and correlates with worse patient outcomes [69–71]. nEGFR has been shown to drive a growing list of oncogenes when associated with transcription factors in the nucleus, as well as modulate the epigenetic landscape to favor tumor progression [56–58, 60–62, 65, 66, 68]. The nuclear localization of EGFR has been shown to correlate with worse prognosis in an ever growing list of cancer including, breast [71], colorectal [70], ovarian [72], lung [73], oropharyngeal [74] and laryngeal [75]. Importantly, these studies demonstrate that nEGFR levels are a better predictor of outcomes than surface or whole cell EGFR levels. This presents an opportunity to develop therapeutics to block the nuclear functions of EGFR, to make clinical impact in TNBC and other tumors where EGFR undergoes retrograde trafficking.

Several therapeutic agents have been developed to target the retrograde trafficking of EGFR, primarily by blocking one of several protein interactions which drive this trafficking. At the plasma membrane in unpolarized epithelia during tumor progression, EGFR forms a complex with MUC1. This complex initiates a series of events that drive EGFR retrograde trafficking and has been targeted with several peptide-based inhibitors. These therapeutic peptides typically mimic some portion of the binding interface to act as a competitive inhibitor once introduced to the cell. However, therapeutic peptides are typically too large and charged or polar to be readily cell permeable. To allow cell penetration, a protein transduction domain is added to these peptides, allowing them to freely enter the cell [76].

The Schroeder lab and others have previously produced therapeutic cell penetrating peptides that target the interaction of EGFR and MUC1. The first peptide generated, PTD4-MUC1-inhibitory-peptide (PMIP), based on the cytoplasmic domain of MUC1 which interacts with EGFR, initiating its retrograde trafficking [77] (Table 1). PMIP was shown to reduce EGFR levels in cell culture and in the mouse mammary tumor virus-driven polyoma middle T-antigen (MMTV-PyV MT) mouse model of breast cancer. Along with the reduction of EGFR levels, a reduction in tumor growth and an induction of caspase3 cleavage was observed, suggesting these tumors were undergoing apoptotic cell death. Clinical translation of this peptide was not possible, as it proved to be unstable in high concentrations (data not shown). Another series of peptides were generated to target the cytoplasmic domain of MUC1 called GO-201 [78], GO-202 [78], GO-203 [79] (Table 1). These peptides mimicked regions of the intracellular domain and induced tumor regression in several xenograph models of breast, prostate cancer [80], and NSCLC [81]. The GO-203 peptide was then reformulated into a nanoparticle containing several 203 peptides called GO-203-NP which increases efficacy of GO-203 by increasing the cellular uptake of GO-203 [82]. Phase 1/2 clinical trial results demonstrate efficacy for GO-203-2C, a peptide that blocks MUC1 dimerization, in combination therapy with decitabine in acute myeloid leukemia (AML) patients [83].

**Table 1:** Theraputic peptides targeting protiens required for EGFR retrograde trafficking

Name	Peptide mimetic	Mechanism of action	Reference
PMIP	MUC1 c-tail	EGFR-Muc1 binding	Bitler et al. [77]
GO-201	MUC1 c-tail	Binds Muc1 c-tail	Raina et al. [78]
GO-202	MUC1 c-tail	Binds Muc1 c-tail	Raina et al. [78]
GO-203	MUC1 c-tail	EGFR-Muc1 binding	Kharbanda et al. [79]
GO-203-NP	MUC1 c-tail nano particle	Increased cellular uptake of GO-203 peptide	Hasegawa et al. [82]
EJ-1	EGFR JXM domain	EGFR dimerization, nuclear localization, calmodulin binding, basolateral targeting, mitochondrial localization	Hart et al. [84]
SAH5	Hydrocarbon stapled EGFR JXM domain	EGFR dimerization, nuclear localization, calmodulin binding, basolateral targeting, mitochondrial localization	Maisel et al. [85]
cSNX1.3	SNX1 Bar domain	EGFR-SNX1 binding	Atwell et al. [86]

Peptides derived from MUC1 cytoplasmic tail, EGFR juxtamembrane (JXM) domain, and SNX1 have demonstrated anti-tumor effects in models of breast, prostate, glioblastoma, and lung cancer. PMIP, EGFR JXM-1 (EJ-1), stapled aromatic hydrocarbon EJ1-5 (SAH5), and cSNX1.3 have all been shown to directly inhibit the retrograde trafficking of EGFR. The GO peptides have been shown to inhibit the nuclear accumulation of MUC1 and inhibit the retrograde trafficking of EGFR

Alternatively, an EJ-1 was generated to interfere directly with the nuclear localization sequence of EGFR [84] (Table 1). The JXM, region of EGFR, is a significant regulator of EGFR biology as it contains a nuclear localization sequence [87], calmodulin-binding domain [88], dimerization domain [89], and basolateral targeting domain [90]. Not only is this region critical for EGFR regulation, but it is also structured as an alpha-helix, and the resulting EJ-1 peptide was far more stable than PMIP. To increase the stability further, this peptide was stapled by substituting 2 residues with non-natural amino acids that were covalently linked through a hydrocarbon linker arm [85]. The resulting stapled peptide, SAH5, had greater stability and was more potent than EJ-1 (Table 1). SAH5 significantly reduced the growth of SUM149, an inflammatory TNBC cell line, xenographs in severe combined immunodeficiency (SCID) mice. However, this peptide was shown to have toxic side effects that included injection site irritation, constriction of veins upon injection, and the induction of seizures in animals shortly after injection (data not shown). Cell culture analysis demonstrated that this peptide induced the opening of calcium channels and altered subsequent calcium signaling (data not shown). These off-target effects increased the toxicity to a level that prevented clinical translation of this peptide.

Recently, the Schroeder lab targeted the endosomal trafficking protein SNX1 to generate a peptide that mimics the interaction domain between EGFR and SNX1, called cSNX1.3 (Figure 1, Table 1) [86]. This end-capped peptide directly binds EGFR with a similar binding efficiency as SNX1 and results in a significant reduction of nEGFR in TNBC cells with amplified EGFR. cSNX1.3 treatment induces an EGFR-dependent impact on oncogenic phenotypes including proliferation, survival, migration, and mammosphere formation while having no impact on immortalized breast epithelial cells. In the transgenic mouse model whey acidic protein-transforming growth factor α (WAP-TGFα), which develops EGFR-dependent mammary carcinomas, 1/3 of animals showed partial regression, 1/3 no progression and 1/3 demonstrated a complete regression with no observable tumor upon necropsy. Further analysis demonstrated no toxicity and circulation of cSNX1.3 was found up to 24 h after injection, indicating this may be a viable therapeutic (data not shown).

Of note, in cell culture experiments, cSNX1.3 had no effect on EGFR signal transduction, internalization, or degradation. This indicates that results observed were not due to an effect on canonical activities of EGFR. Rather, cSNX1.3 seemed to only effect the retrograde trafficking of EGFR, further highlighting the importance of this pathway for the understanding and treatment of cancer. These and other peptide-based therapeutics that block protein-protein interactions hold promise for future clinical therapeutics.

## Conclusions

EGFR is a complex protein that plays roles in many aspects of tumor growth and metastasis. Many drugs have been designed to target well characterized aspects of EGFR biology like ligand binding and kinase signaling. TKIs and antibody-based therapies have significantly improved many patient outcomes over several decades. However, tumors often develop resistance to these drugs either through activation of other signal transduction pathways or through additional mutations. In cases where there is an abundance of nEGFR these drugs don’t often work. nEGFR is a major driver of tumor progression, metastasis, and resistance to anti-EGFR therapies and has been suggested to be a better biomarker than total cell EGFR levels.

The tumor specificity of nEGFR makes it a potentially valuable therapeutic target as nEGFR is rarely seen outside the context of cancer. This tumor specificity is in stark contrast to the roles of EGFR’s kinase domain which are critical in a majority of human tissues at nearly all developmental stages. The reliance on EGFR to develop and maintain human tissues, including the skin and gastrointestinal (GI) tract, explains many side effects seen with anti-EGFR therapies including rash, nausea, and diarrhea.

As has been demonstrated with several peptide inhibitors of EGFR retrograde trafficking, nEGFR is a druggable target. The trafficking of EGFR to the nucleus requires several protein interactions that alter the posttranslational modification seen on EGFR, leading to a switch from lysosomal degradation to nuclear trafficking. The interactions of EGFR with MUC1 and SNX1 have both been targeted and shown efficacy in animal mouse tumor models. The interaction of EGFR and MUC1 is an upstream step in the nuclear trafficking of EGFR and might induce the degradation of EGFR in addition to the loss off nuclear localization making it an ideal anti-nEGFR therapy. In contrast targeting a nEGFR trafficking protein, like SNX1, is further downstream and is unlikely to induce EGFR degradation. However, targeting SNX1 might have additional benefits that have not been fully characterized. SNX1 binds EGFR on the kinase domain and induces the retrograde trafficking of EGFR. SNX1 also binds several other RTKs that have also been shown to drive oncogenesis with their nuclear activities. The peptide cSNX1.3 which was designed to block the interaction of EGFR and SNX1 inhibited 2 dimensional (2D) cell migration driven by EGFR. cSNX1.3 also inhibited migration driven by HER3 and c-Met, which are both RTKs shown to interact with SNX1. They undergo nuclear translocation and drive oncogenesis from within the nucleus suggesting the nuclear localization of RTKs more broadly could be a target for therapeutic intervention and targeting sorting proteins could be an avenue to a broad acting anti-RTK retrograde trafficking therapy.

## Abbreviations

ADCs: antibody-drug conjugates
cSNX1.3: capped sorting nexin peptide 1.3
EGFR: epidermal growth factor receptor
EJ-1: epidermal growth factor receptor juxtamembrane-1
HER: human epidermal growth factor receptor
MUC1: mucin-1
JXM: juxtamembrane
nEGFR: nuclear epidermal growth factor receptor
NK: natural killer
NSCLC: non-small cell lung cancer
PMIP: PTD4-mucin-1-inhibitory-peptide
PX: phox homology
RTKs: receptor tyrosine kinases
SAH5: stapled aromatic hydrocarbon epidermal growth factor receptor juxtamembrane-1
SNXs: sorting nexins
STAT: signal transducer of activators of transcription
TKIs: tyrosine kinase inhibitors
TNBC: triple negative breast cancer

## Declarations

### Copyright

© The Author(s) 2023.
